# Nitrogen cycling in pastoral livestock systems in Sub‐Saharan Africa: knowns and unknowns

**DOI:** 10.1002/eap.2368

**Published:** 2021-06-10

**Authors:** Victoria Carbonell, Lutz Merbold, Eugenio Díaz‐Pinés, Thomas P. F. Dowling, Klaus Butterbach‐Bahl

**Affiliations:** ^1^ Department of Environmental System Sciences Institute of Agricultural Sciences, Grassland Science Group ETH Zurich Universitaetsstrasse 2 Zurich 8092 Switzerland; ^2^ Mazingira Centre International Livestock Research Institute (ILRI) PO Box 30709 Nairobi Kenya; ^3^ Karlsruhe Institute of Technology Institute of Meteorology and Climate Research Atmospheric Environmental Research (IMK‐IFU) Kreuzeckbahnstraße 19 Garmisch‐Partenkirchen 82467 Germany; ^4^ Agroscope, Research Division Agroecology and Environment Reckenholzstrasse 191 Zurich 8046 Switzerland; ^5^ Institute of Soil Research (IBF) University of Natural Resources and Life Sciences (BOKU), Vienna Peter‐Jordan‐Straße Vienna 82 1190 Austria; ^6^ Department of Geography Kings College London Bush House (NE), 30 Aldwych London WC2B 4BG United Kingdom

**Keywords:** ammonia, greenhouse gas emissions, knowledge gaps, leaching, pastoralism, rangelands, savanna

## Abstract

Pastoral systems are the dominant livestock production system in arid and semiarid regions of sub‐Saharan Africa (SSA). They are often the only form of agriculture that can be practiced due to unfavorable climate and soil fertility levels that prevent crop cultivation. Pastoralism can have negative impacts on the environment, including land degradation, greenhouse gas emissions and other gases to the atmosphere, soil erosion, water pollution and biodiversity loss. Here, we review the current knowledge on nitrogen (N) cycling, storage, and loss pathways, with an emphasis on identification of N emission hotspots. Our review reports a large uncertainty in the amount of N lost as ammonia from excreta and manure storage, as well as N losses via nitrate and DON leaching. We also found that another major N loss pathway (18%), soil N_2_ emissions, has not yet been measured. In order to summarize the available information, we use a virtual pastoral farm, with characteristics and management practices obtained from a real farm, Kapiti Research Station in Kenya. For outlining N flows at this virtual farm, we used published data, data from global studies, satellite imagery and geographic information system (GIS) tools. Our results show that N inputs in pastoral systems are dominated by atmospheric N deposition (˜80%), while inputs due to biological nitrogen fixation seems to play a smaller role. A major N loss pathway is nitrogen leaching (nitrate > DON) from pastures (33%). Cattle enclosures (bomas), where animals are kept during night, represent N emissions hotspots, representing 16% of the total N losses from the system. N losses via ammonia volatilization and N_2_O were four and three orders of magnitude higher from bomas than from the pasture, respectively. Based on our results, we further identify future research requirements and highlight the urgent need for experimental data collection to quantify nitrogen losses from manure in animal congregation areas. Such information is needed to improve our understanding on N cycling in pastoral systems in semiarid regions and to provide practical recommendations for managers that can help with decision‐making on management strategies in pastoral systems in semiarid savannas.

## Introduction

In developing countries, population growth, rising incomes and urbanisation have caused an increase in the demand for protein rich food, such as meat and dairy products (Swanepoel et al. 2010). Driven by this demand, livestock in developing countries is one of the agricultural subsectors experiencing the fastest economic growth, with a 33% share of agriculture gross domestic product. An intensification of livestock production is particularly expected in Africa, as it is the only continent with a predicted positive population growth trend for the next 80 yr (FAO 2013). At the same time, approximately 25% of the global cattle population can be found on the African continent (Robinson et al. 2011). Livestock herding is often the primary, if not the only, source of income for the rural local populations (Barrett et al. 2003). Around 300 million people, 23% of the world’s poor live in sub‐Saharan Africa (SSA), of which 60% are known to depend on livestock for their livelihoods (Thornton et al. 2002). The negative impacts of climate change in Africa, among political, social, and economic challenges, increase vulnerability of pastoral systems (FAO 2018). A better understanding of dryland dynamics has led to a new rangeland paradigm (Turner 2011), which recognizes that pastoralism, through mobility as its adaptation to extreme climatic conditions, is the most productive and sustainable use of these lands. This represents a change in the conception of pastoralism and debates “the tragedy of the commons” (Hardin 1968), which blames pastoralism for desertification (FAO 2018). Keeping livestock is an important strategy to improve food security and wellbeing by providing nutrition and generating income from products and services (e.g., draft power for transport and tillage; Stroebel et al. 2008, Thornton 2010). It also represents an important contribution to some African communities’ cultural beliefs and practices (Horsthemke 2017). In SSA, the increase of consumption per‐capita is anticipated to be 32% for meat and 17% for milk between 2000 and 2030 (Robinson and Pozzi 2011). The change of consumption patterns in favor of livestock products offers the opportunity for poor livestock farmers to generate additional income (Thornton 2010), creating further demand for intensification. However, the change from pastoralism to sedentary modes of cattle farming may result in overgrazing and land degradation.

With decreasing soil fertility and increasing aridity, intensification of livestock production will likely be strongly affected by climate change. This is part of a negative feedback cycle given that livestock production is a major source of greenhouse gases (GHG) and thus contributes to climate change (Tubiello et al. 2013). More generally, livestock production systems (LPS) entail negative environmental consequences that are linked to changes in landscape and ecosystem nitrogen (N) cycling (Galloway et al. 2010). These consequences include land degradation, air and water pollution due to the emission of ammonia (NH_3_) from excreta and nitrogen losses along hydrological pathways, contamination of surface waters with pathogens and nutrients, and losses in biodiversity (Steinfeld et al. 2006).

In view of the growth trend of livestock production in SSA and its tremendous potential effects on the environment, there is a strong need for livestock production intensification to go hand in hand with appropriate management. This can partially be achieved through N cycling monitoring (e.g., farm inventories to identify major N pools and flows; Van den Bosch et al. 1998), as intensification will change N dynamics drastically. Subsequently, evidence‐based mitigation and adaptation strategies can be developed by managing N to reduce known negative impacts of livestock systems on the environment (e.g., improved manure collection and storage to avoid nutrient losses), without affecting the additional income and food security for livestock keepers (Thornton et al. 2009).

Pastoralism is the dominant livestock‐keeping form in African drylands, occupying at least 40% of the continent’s land mass (FAO 2018). In SSA alone, livestock is the primary source of income for 25 million pastoralists (>50% gross revenue comes from livestock or livestock‐related activities; Swift 1988) and 250 million agro‐pastoralists (>50% gross revenue comes from agriculture and >10% from livestock; Swift 1988). Despite the socioeconomic importance of pastoral systems in SSA, very little research has focused on N cycling in such systems. While there is information available on N dynamics in arid and semiarid lands, such as atmospheric N budget studies (Delon et al. 2010), a comprehensive assessment of N cycling in pastoral systems is yet lacking. Robust N budgeting is challenging as both the abundance and the spatial distribution of N in pastoral systems are strongly affected by management practices and climatic conditions (Dahal et al. 2018). Therefore, in this paper, we aim to (1) identify and summarize existing knowledge on N cycling in pastoral systems in arid and semiarid SSA, (2) estimate the contribution of N input and N losses to the total N budget of a pastoral farm, as well as N pools and N translocation within the farm, and (3) identify gaps and constraints that might be impeding a realistic environmental impact and N management assessment of these LPS in SSA.

## Characterization of Pastoral Livestock Systems in Sub‐Saharan Africa

Pastoralism in SSA is a traditional LPS that can be mainly found across the savanna belt in arid, semiarid, (Koppen‐Geiger climates hot desert [BWh] and hot semiarid [BSh] regions; Kottek et al. 2006). The practice often represents the only viable means of agriculture in these regions. It occupies 346 million ha in SSA (i.e., 14% of the SSA land; Dixon et al. 2001). Pastoralism in SSA is characterized by a set of unique management practices, where daily and seasonal mobility of livestock is a key feature. In their daily movements, cattle graze during the day in the savanna and are taken to drink in the morning and in the evening, before being grouped overnight in cattle enclosures (*boma* in Swahili; *kraal* in Afrikaans). Thus, nutrients are translocated as a result of cattle grazing the savanna and excreting manure on pasture and in congregation areas, such as in bomas and around waterholes (Butterbach‐Bahl et al. 2020). Pastoral systems in SSA often consist of three distinct areas: (1) rangeland, occupying >96–98% of the landscape area, (2) animal enclosures (i.e., bomas) and surroundings, and (3) water points that include wells and rivers. The concentration of animals in congregation areas causes high accumulation of manure (Tolsma et al. 2018), as well as alteration of the vegetation patterns (Andrew 1988, Augustine 2003, Muchiru et al. 2009). Areas with a localized impact on vegetation and soil structure caused by livestock are called “piospheres” (Andrew 1988). Furthermore, continuous cattle trampling on pathways and in congregation areas leads to soil compaction and reduction in soil fertility. The loss in fertility occurs due to a reduction in pore size and thus water infiltration, factors that negatively affect both soil hydraulic properties and soil water and nutrient storage (Hamza and Anderson 2005).

Key management practices and features of pastoral systems in SSA with regard to their effects on N dynamics can be summarized as (1) redistribution of N through grazing and deposition of feces across the landscape during the daytime, (2) Accumulation of N (manure) in bomas, where livestock herds are held overnight, (3) accumulation of N (livestock/wildlife manure) around watering points, and (4) additional regional redistribution of N due to seasonal movements of herds.

Nitrogen inputs into the system occur through biological nitrogen fixation (BNF), wet and dry deposition of reactive N, e.g., ammonia (NH_3_) and nitrogen oxide (NO_x_, NO + NO_2_), to the land surface as well as via supplementary feed (if provided). Nitrogen losses from pastoral systems occur either in gaseous form, e.g., NH_3_ volatilization, nitrous oxide (N_2_O), NO_x_ or dinitrogen (N_2_) emissions, or along hydrological pathways due to leaching or surface run‐off of organic and inorganic N compounds, e.g., nitrate (${\text{NO}}_{3}^{‐}$), dissolved organic nitrogen (DON), and ammonium (NH_4_^+^; Carran and Clough 1996).

Gaseous N losses, as well as leaching, occur in all three areas with varying magnitude. Other N losses occur through export of animal products (meat/milk) and occasional fires. Congregation areas are expected to have higher gaseous and hydrological N losses, specifically in the form of N_2_O, N_2_, NH_3_, and NO_x_ emissions and ${\text{NO}}_{3}^{‐}$ leaching, than the surrounding landscape, as a result of N concentration effects.

## Methodology

### Search protocol and selection criteria

To characterize and quantify N dynamics and environmental N losses of pastoral systems in SSA a literature review was carried out using available databases (Scopus, Web of Knowledge) and search engines (Google Scholar). We used generic keywords (nitrogen, savanna, Africa), in combination with specific keywords associated to N flows and stocks (Appendix S1: Table S1), for identifying publications that study N in pastoral systems, in SSA savannas. We selected data from publications from arid and semiarid lands (ASAL) in SSA which either reported on N flows or allowed us to estimate N fluxes or pools from measurements of biomass and C:N ratios. All data were converted to kg N·ha^−1^·yr^−1^ for N fluxes or kg N/ha for N stocks.

Data retrieved from the search were classified into four categories for further analysis: (1) soil N stocks, (2) plant N stocks, (3) N inputs and export/losses into and from the landscape, and (4) N emissions from a priori hotspots of transformations, i.e., bomas and piospheres. When specific information on SSA pastoral systems was not available, we also considered global databases (e.g., soil database ISRIC‐WISE), and emission factors available within the IPCC guidelines for national greenhouse gas inventories (IPCC 2019). However, where there was still missing information, e.g., on soil N_2_ fluxes from savanna, we made a best estimate judgement (Appendix S1: Fig. S1).

### Data collection, calculation, and error propagation

To better illustrate N dynamics in pastoral systems, we made use of a virtual ranching system. For this, we considered the features of livestock management, vegetation, soils, and climate of Kapiti Research Station (Table 1), a ranching system located in a semiarid region in Southern Kenya (1°37′49.908″ S, 37°8′43.195″ E). The climate is typical for semiarid savannas, with precipitation below potential evapotranspiration. The mean annual precipitation is 550 mm, with rainfall being distributed in a bimodal precipitation regime, in March–May (CV = 0.16) and October–December (CV = 0.09; McCown and Jones 1992, Berliner and Kioko 1999). Approximately 80% of the annual precipitation occurs during these two periods. The mean annual temperature is 20.2°C, with 4°C of annual variation.

**Table 1:** Input data to calculate input/output N balances of a virtual pastoral farm in Kenya.

Variables	Units	Value	Reference
Farm area	ha	10,000	I. Gluecks, *personal communication*
Number of cattle	TLU‡	1,858	interview†
Stocking rate	TLU/ha	0.19	
Number of bomas in use	#	19	interview
Number of cattle/boma	TLU	98	
Area of a boma§	ha	0.03	interview
Time in boma/day	d	0.5	interview
Number of waterholes in use	#	5	interview
Time at waterholes/day	d	0.1	interview
Area of a waterhole	ha	1	interview
Piosphere area¶	ha/waterhole	6.7	Smet and Ward (2006)
Dry season duration	d	240	
Wet season duration	d	120	
N excretion dry season#	g N·TLU^−1^·d^−1^	101 ± 30	Schlecht et al. (1995)
N excretion wet season#	g N·TLU^−1^·d^−1^	129 ± 31	Schlecht et al. (1995)
N intake dry season#	g N·TLU^−1^·d^−1^	102 ± 30	Schlecht et al. (1995)
N intake wet season#	g N·TLU^−1^·d^−1^	147 ± 44	Schlecht et al. (1995)
N excretion lost as N_2_O from manure in bomas	%	0.14	Zhu et al. (2018)
N excretion lost as N_2_O from manure left on pastures	%	0.2 (0–0.6)	IPCC (2019)
N excretion lost as NH_3_ and NO_x_ from manure in bomas	%	30 (20–50)	IPCC (2006), Delon et al. (2010)
N excretion lost as NH_3_ and NO_x_ from manure left on pastures||	%	15.3	IPCC (2019)
N excretion lost as ${\text{NO}}_{3}^{‐}$ leaching	%	3.5 (0–7)	IPCC (2019)
N lost as DON leaching††	%	30	Siemens and Kaupejohann (2002), Wachendorf et al. (2005), Cai and Akiyama (2016)
N excretion lost as N_2_ ‡‡	%	6 (1–10)	Jarvis and Pain (1994), IPCC (2019)

† Interview in November 2018 with the Kapiti Farm and Research manager, and farm veterinarian.

‡ TLU refers to Tropical Livestock Unit of 250 kg live mass (Schlecht and Hiernaux 2005), equivalent to 1.4 head of cattle. The number of cattle reported in ILRI Kapiti Research Station was 2,600, ˜1,858 TLU.

§ Calculated from an average boma diameter of 20 m.

¶ The piosphere area was calculated assuming a radius of 100 m around the waterhole (Smet and Ward 2006).

# N excretion and intake values for dry and wet season.

|| Median value. Most N volatilized (˜93%) occurs in form of NH_3_, the rest (7%) is estimated to occur as NOx.

†† Percentage of total N leached for manure and soils.

‡‡ N_2_ losses from manure are estimated to be three times greater than those as N_2_O (Jarvis and Pain 1994).

Cattle management on the farm is typical for pastoral systems, where herders graze animals during the day and enclose them in bomas during the night. There are bomas distributed in different areas of the farm, and each area is used for a few months (two to three) every year, until cattle are moved when the pasture in the area surrounding the bomas is exhausted. Some of the bomas are subject to occasional extraction of manure by local farmers.

Soils on the farm plains are Vertisols (Chesworth 2008), dark clayey soils locally known as “black cotton soils,” combined with higher areas of Nitisols. Some areas are dominated by savanna grasses (e.g., *Themeda*, *Panicum*), whereas, in other areas, diverse densities of *Vachellia drepanolobium* and *Balanites glabra* are found.

Such ranching systems usually differ in a few aspects from a traditional pastoral system as there is clear boundary delimitation, and improved management with regard to herd composition, pasture, and watering points (Otte and Chilonda 2002). However, the overall system function is similar to other LPS in SSA, and this type of system can be found in semiarid areas of West, East, and Southern Africa. The calculation of input/output N balance of the virtual farm was carried out in three steps: (1) nitrogen fluxes in savanna soil, (2) nitrogen fluxes in potential “hotspots” (bomas and waterholes), and (3) contribution of each N flow to the total N budget taking into account the area occupied by bomas and waterholes within the farm.

#### Soil and vegetation nitrogen stocks and tree density

Soil N stocks for 0–100 cm depths were calculated by extracting data from the Africa soil profiles database (Leenaars 2013), available at the International Soil Reference and Information Centre (ISRIC) website (*available online*).^8^ Soil profiles for this study were selected by overlapping soil profiles GPS coordinates from the database with a GIS shapefile of global pastoral systems (i.e., arid/semiarid rangeland based, LGA; Seré and Steinfeld 1996; Appendix S1: Fig. S2). We determined pastoral regions in SSA on basis of the *Global livestock production systems 2007 v.3 map* from the FAO‐Geonetwork website (*available online*).^9^

The overlapping soil profiles were selected, and the soil type was assigned to each of these points by overlaying them with the FAO/UNESCO Soil Map of the World (Food and Agriculture Organization of the United Nations 1974), using shapefiles from the FAO‐Geonetwork (see footnote 9). N stocks for the selected points for 0–100 cm depth were calculated according to Ellertl and Bettany (1995)

(1) $$M_{\text{nitrogen}}=\text{conc}\times \rho_{b}\times d\times 10,000\;m^{2}/\text{ha}\times 0.001\;\text{Mg/kg}$$

where *M*_nitrogen_ is the N mass per unit area (Mg N/ha), conc is the N concentration (kg/Mg), and ρ_b_ is the soil bulk density (Mg/m^3^). To estimate vegetation N stock values of our virtual farm we first assessed the percentage of savanna that is covered by vegetation (grasses and trees). For this, we used Sentinel 2 multiband (RGB‐VNIR) imagery over Kapiti Research Station to carry out a supervised land surface classification using a Random Forest (e.g., Phan and Kappas 2018). Canopy vegetation is here defined as trees and large shrubs that grow from a central trunk and branch out to shade the ground beneath. The RGB‐VNIR bands are downloaded from the Sentinel Hub website with atmospheric correction and georectification pre‐applied and then stacked into a single raster (*data available online*).^10^ Training data were manually constructed for the farm by the drawing of polygons around known pixel types. A pixel type was identified by overlaying high‐resolution imagery from the Google WMS satellite service (Quick Bird imagery) on the Sentinel 2 raster. This allowed us to map which pixels contained canopy vegetation and which contained other vegetation classes. As the focus here was to find the percentage cover of canopy vegetation and other vegetation, the classes settled upon were (1) canopy vegetation, (2) exposed soil (including tracks and unmetalled roads), (3) other vegetation, and (4) water bodies. The Random Forest algorithm was then trained on this manually generated set of pixel classifications and applied to the whole scene. The result of the classification was a tree/shrub canopy cover over Kapiti Ranch of 9%, grassland cover of 80%, and bare soils of 11%.

#### Nitrogen inputs and exports/losses

Atmospheric N deposition data were extracted from the global maps of atmospheric N deposition, 1860, 1993, and 2050 database (Dentener 2006). In this database, N deposition estimates were calculated with a resolution of 5° longitude by 3.75° latitude. We projected model estimates from 1993 across arid and semiarid pastoral regions of SSA (Appendix S1: Fig. S2) with QGIS Development Team (2017), resulting in 54 pixel values.

NH_3_ and N_2_O emissions from the grazing area, bomas, and piospheres were related to the number of head of cattle following the methodology by Delon et al. (2010) (Appendix S1: Fig. S3). The daily amount of total N excreted by cattle, defined as g N per Tropical Livestock Units per day (g N·TLU^−1^·d^−1^), was calculated from Schlecht et al. (1995) for both wet and dry seasons. The time spent by the cattle in the three areas as reported by the farm manager (i.e., 50%, 40%, and 10% in grazing areas, bomas, and around waterholes, respectively, Table 1) was used to estimate the amount of total N excreted per TLU on each of the areas. This number was then multiplied by the cattle stocking rate (TLU/km^2^) for the grazing areas, bomas or piospheres, estimated in our virtual farm using the GLiPHA (Global Livestock Production and Health Atlas) database (Table 1).

A loss rate of 30% (IPCC 2006) of the N excreted was applied for NH_3_ volatilization from bomas, and 15.3% (IPCC 2019) of the N from manure left on pasture; we used 0.14% as N lost in form of N_2_O from excreta on tropical pastures in Kenya (Zhu et al. 2018) (Table 1). Annual NH_3_ and N_2_O emissions were calculated for dry and wet seasons with different N excretion rates, by multiplying daily emissions with the number of days of the respective seasons

(2) $$\text{Flux}=N_{\text{ex}}\times t_{f}\times \rho \times L_{r}\times t\times 1,000/100$$

where Flux is the NH_3_ or N_2_O flux in kg N·ha^−1^·yr^−1^, N_ex_ is the amount of N excreted by cattle in g N·TLU^−1^·d^−1^ in the dry or wet season, *t_f_* is the percentage of time spent in savanna, bomas, or piospheres, ρ is the stocking rate in savanna, bomas or piospheres in TLU/ha, *L_r_* is the percentage N loss rate for NH_3_ or N_2_O, and *t* is length of the dry or wet season in d (Table 1).

#### Nitrogen balance of the virtual farm

To fulfil the second aim of this study, we hypothesized a ranching system, using the attributes from the Kapiti Research Station, in Southern Kenya. The advantage of using Kapiti Research Station is reliable access to accurate information on a wide range of variables provided by the farm manager (Table 1). To calculate the contribution of each N flow to the overall N budget, estimated N flows are multiplied by the source area and divided by the total farm area (i.e., 10,000 ha).

#### Error propagation

Each of the numbers provided in this review shows the current knowledge on the error involved. The errors were calculated as follows: If the errors were given in the respective literature and more than one publication was available, we took the mean of the stated errors. In the cases where only a single publication was available, we state the respective value and indicate this specifically. If no error estimates were provided, we assume an uncertainty of 50%. We further highlight whether we show SE or SD.

## Results and Discussion

Several publications have studied N cycling in tropical savannas in South Africa (Coetsee et al. 2012), West Africa (Delon et al. 2010), Australia (Holt and Division 2018) and America (Bustamante et al. 2006). However, to the best of our knowledge, to date no study has presented a full N balance including livestock‐related flows from bomas and piospheres. There are studies looking at cattle congregation areas, but they are limited to the exploration of differences in soil N and vegetation inside cattle bomas and its surroundings in pastoral systems (Reid and Ellis 1995, Young et al. 1995, Augustine 2003, Muchiru et al. 2009, Porensky and Veblen 2015, Valls Fox et al. 2015. Young found that vegetation cover (e.g., *Digitaria* sp., *Portulaca oleracea*) was up to 24 times higher in former boma areas than in the surroundings several decades after boma abandonment. They also found that soil pH and N content decreased with increasing distance from the bomas. We were not able to find any study that estimates GHG emissions in bomas in pastoral systems in SSA and its relevance for farm and regional N budgets.

### Nitrogen stocks

#### Soil N stocks

Savannas are patchy environments due to differences in soil nutrient content, human and animal activities, and their productivity greatly varies across SSA as a result of different soil fertility and rainfall patterns, two key features that determine plant productivity (Scholes and Walker, 1993). There are a limited number of studies on nutrient balances in Africa that include N stocks (Cobo et al. 2010), even though soil fertility has a great effect on savanna ecology, influencing the proportion of primary production converted to herbivore biomass, thus strongly controlling the N cycle (Scholes and Walker, 1993). Studies that reported on soil N stocks in savannas only considered the top 10–30 cm of the soil profile (Bernhard‐Reversat 1982, Scholes and Andreae 2000, Lesschen et al. 2007, Cech et al. 2010). However, in fertile savannas, subsoil (>30 cm depth) can contribute to more than half of the total soil C stocks and therefore needs to be considered in C (and N) cycle analysis (Rumpel and Kögel‐Knabner 2011). Here, we present soil N stocks estimations in pastoral regions in SSA down to 1 m depth, integrating information from 178 profiles across SSA. Different soil types with different fertility status were included in our estimations, predominantly Arenosols, Regosols, and Vertisols (Appendix S1: Fig. S2, Table S2). The average soil N stock in 0–100 cm in SSA savannas was 5.0 ± 3.6 Mg/ha (mean ± SD), which is similar to what has been reported for the South American Cerrado savannas, (e.g., 4.6 Mg/ha in Brazil and 5.8 Mg/ha in Venezuela (Bustamante et al. 2006).

In pastoral systems, tree‐grass spatial patterns affect soil organic matter distribution and thus N availability. Higher soil N has been found beneath *Acacia s.l*. (divided into two distinct genera for African species since 2011, *Vachellia* sp. and *Senegalia* sp., Kyalangalilwa et al. 2013), and *Balanites*, compared to the open areas (Bernhard‐Reversat and Poupon 1980, Treydte et al. 2008). This is due to the capacity to fix N symbiotically that is present in many tree species in the African savanna, predominantly from the Fabaceae family (Cech et al. 2010). Soil N is further expected to be higher under the canopy due to the effect of shade reducing soil temperature and therefore decomposition of organic matter, as well as increased litterfall and wildlife presence under the trees (Simón et al. 2013). This suggests that soil N distribution in savannas is patchy and that besides the influence of soil particle distribution (larger N pool in fine‐textured soils than in coarse‐textured soils; Jacobs et al. 2007), spatial distribution of vegetation should be considered when estimating soil N stocks. We used the N stocks average value of Arenosols for the open areas of our virtual farm (predominant savanna soil in semiarid SSA, covering 21% of total area). We estimated canopy cover to be 9.0% (Appendix S1: Table S3), and following the higher concentrations under trees, where soil N stock is double (i.e., 9.8 ± 8.0 Mg/ha; Bernhard‐Reversat 1982), calculated a total soil N stock of 5.3 ± 4.3 Mg N/ha (Table 2).

**Table 2:** Soil N stocks and vegetation N stocks for pastoral systems in sub‐Saharan Africa (SSA).

Ecosystem compartments	Stock	References
Soil N stock (0–100 cm) in open areas (Mg N/ha)	4.9 ± 4.0	calculated from Leenaars (2013)
Soil N stocks underneath trees (Mg N/ha)	9.8 ± 8.0†	Bernhard‐Reversat (1982)
Total soil N stocks (Mg N/ha)	5.3 ± 4.3	–
Grass biomass (kg/ha)	2,980‡	Wang et al. (2012)
Grass N content (% DM)	0.53§	Knox et al. (2011)
Total Grass N stock (kg N/ha)	12.6¶	–
Tree N stocks (kg N/(ha of canopy cover))	301.7 ± 35.6#	Bernhard‐Reversat and Poupon (1980)
Tree N stock (kg N/ha)	27.0 ± 3.2||	–
Total N vegetation stock (grass + trees) (kg N/ha)	39.6 ± 3.2	–
Total N stock (soil + vegetation) (Mg N/ha)	5.4 ± 4.4	–

Notes

Values are mean ± SD. DM, dry mass.

† Calculated assuming soil N stock doubles in the area underneath the trees to be applied in 9% (900 ha) canopy cover calculated from satellite imagery.

‡ Value for grazed pasture in a Namibian savanna.

§ Value from grass samples in a South African savanna.

¶ Value calculated for 80% cover (i.e., 8,000 ha) calculated from satellite imagery.

# Mean value from 12 *Senegalia senegal* samples in a Sahelian savanna.

|| Calculated for 9% canopy cover (i.e., 900 ha) calculated from satellite imagery.

#### Vegetation N stocks

Following soil, N stored in the vegetation represents the second largest N pool in a pastoral system (Sarmiento 1984). However, accurate estimates are difficult to derive due to the different vegetation distribution of trees and grasses across the arid and semiarid regions and within respective livestock systems. Typical vegetation in SSA pastoral systems is composed by a grass layer (e.g., *Themeda* genus*),* dispersed trees (e.g., *Vachellia* and *Senegalia* genera) and shrubs (e.g., *Euphorbia* sp. and *Hibiscus* sp.). We used aboveground grass biomass estimates from grazed pasture samples for Kalahari, Namibia (2,980 kg/ha; Wang et al. 2012; Table 2). The N content in grass biomass was assumed to be 0.53% dry matter, DM, i.e., the mean value of N contents for grasses (range 0.31–0.91% DM) in a study carried out in South Africa (Knox et al. 2011). Considering a grass cover of 79.6% (Appendix S1: Table S3), the grass N content in our farm results in 12.6 kg N/ha but expect that the range could be 7.4–21.6 kg N/ha. Our tree N stocks of 27.0 ± 3.2 kg N/ha were estimated with the mean *Senegalia senegal* N content from a study conducted in a Senegalese savanna (Bernhard‐Reversat and Poupon 1980), applied to 9% canopy cover. Total vegetation (trees + grass) N stocks resulted in 39.6 ± 3.2 kg N/ha (Table 2).

### Nitrogen inputs

#### Biological nitrogen fixation

Geographically, tropical savannas have been identified as hotspots for BNF (Vitousek et al. 2013). Nitrogen‐fixing trees, such as *Vachellia tortilis* (Cramer et al. 2009), *Senegalia senegal* (Ndoye et al.1995) and *Balanites aegyptiaca* (Hines and Eckman 1993) are found in fertile tropical savannas, where the optimum temperature of nitrogenase activity (26°C; Houlton et al. 2008) is within the temperature ranges in these regions. In infertile savannas, where there is limited presence of N‐fixing trees, N is added to the soils through herbs species, such as *Crotalaria*, *Indigofera*, and *Tephrosia* genera (Nezomba et al. 2013). Vegetation cover, soil fertility status, edaphic characteristics, water availability, and grazing management vary substantially across savannas, and thus BNF rates vary also. Based on model analysis, Houlton et al. (2008) estimated a global BNF range of 20–60 kg N·ha^−1^·yr^−1^ for tropical savannas. Robertson and Rosswall (1986) report BNF rates of 30 N·ha^−1^·yr^−1^ for a grazed west African savanna, with symbiotic N fixation contributing one‐third to total ecosystem BNF, and non‐symbiotic algal and non‐algal BNF the remaining two‐thirds. Other authors report much smaller BNF values, e.g., 0.4−5 kg N·ha^−1^·yr^−1^ for Sahelian rangelands (Krul et al., 1982), whereas Cleveland et al. (1999) estimated global BNF fluxes of 16.4, 30.2, and 44 kg N·ha^−1^·yr^−1^ for tropical savanna ecosystems with 5%, 15%, and 25% tree cover, respectively. However, this estimate includes fertile and infertile savannas, and has been argued to be too high, due to the upscaling procedure from field‐based measurements (Galloway et al. 2004, Vitousek et al. 2013). Chen et al. (2010) refined estimates from Cleveland et al. (1999) following a bio‐meteorological approach by (Fisher et al. 2008) and obtained a BNF average of 18.6 kg N·ha^−1^·yr^−1^. Cech et al. (2010) estimated BNF from trees (*Vachellia zanzibarica*) and herbs (*Dichrostachys cinerea*) in a nutrient‐poor savanna in Tanzania. Trees and herbs BNF estimations were made using measured biomass production data, tissue N concentrations and proportion of N derived from the atmosphere data from literature. Results of tree BNF ranged from 3 to 12 kg N·ha^−1^·yr^−1^, depending on the stand density, and <0.50 kg N·ha^−1^·yr^−1^ for herbs. We used their reported values of 7.4 ± 4.3 kg N·ha^−1^·yr^−1^ for the tree cover of our virtual farm (9%, i.e., 900 ha, Appendix S1: Table S3), and 0.2 ± 0.1 kg N·ha^−1^·yr^−1^ for other vegetation cover (79.6%, i.e., 7,960 ha, Appendix S1: Table S3), although we expect that the range could be 0.1–60 N·ha^−1^·yr^−1^.

#### Atmospheric N deposition

Atmospheric N deposition (wet and dry) is an N input pathway into ecosystems, but very little information is found in the literature for savanna ecosystems globally, and for SSA in particular. Furthermore, most studies only report wet deposition due to the difficultly in measuring dry deposition. Available wet N deposition estimations in African semiarid savannas range from 2 to 11 kg N·ha^−1^·yr^−1^ (Bate 1981, Ruess and McNaughton 1988, Augustine 2003). With regard to total N deposition, Delon et al. (2012) came up with an estimate of 7.4 ± 1.0 kg N·ha^−1^·yr^−1^ in a dry savanna in West Africa, while Galy‐Lacaux and Delon (2014) observed 6 ± 2 kg N·ha^−1^·yr^−1^ during long‐term measurements (2002–2006) in three savanna sites in Niger and Mali. For our virtual farm, we calculated the mean of total N deposition values in arid and semiarid savannas from the Global maps of atmospheric N deposition for year 1993 (Dentener 2006), resulting in 3.1 ± 2.4 kg N·ha^−1^·yr^−1^ (Table 2), but we expect a range of 0.7–11 kg N·ha^−1^·yr^−1^. Our results are around half of the values from West African savannas, probably because of the great effect of the Harmattan wind on dry deposition in that region (Lesschen et al. 2007). Deposition of reduced N compounds (NO_x_) resulted slightly higher than oxidized N compounds (NO_y_) with averages of 1.7 ± 1.5 and 1.4 ± 1.1 kg N·ha^−1^·yr^−1^, respectively.

### Nitrogen outputs

Nitrogen losses are expected to be higher in congregation areas (i.e., bomas and piospheres) due to enhanced cation exchange capacity with ammonium and potassium, and higher urease activity due to manure accumulation (Sheppard and Bittman 2011), as well as a pH increase (Whalen et al. 2000). Lower C:N ratios are also expected, further contributing to localized N losses in congregation areas. No studies of N losses from livestock enclosures is available for Africa, however a study in semiarid grassland systems of Inner Mongolia showed that sheep enclosures (sheepfolds) are hotspots for NO, N_2_O, and NH_3_ emissions, with mean annual NO emissions of 7.8 ± 1.4 kg N·ha^−1^·yr^−1^ in summer, 17.1 ± 6.1 kg N·ha^−1^·yr^−1^ in winter (Liu et al. 2009), and mean N_2_O annual emissions of 215 ± 35 kg N·ha^−1^·yr^−1^ in summer and 79 ± 26 kg N·ha^−1^·yr^−1^ in winter (Holst et al. 2007). Overall, fluxes of both NO and N_2_O were three orders of magnitude higher in sheepfolds compared to the adjacent grassland. For NH_3_, the highest concentrations were measured close to the sheepfolds (Holst et al. 2007), indicating that they are a major source of this gas.

#### Ammonia (NH3)

A limited number of studies have estimated NH_3_ emissions from pastoral systems in SSA. Grazing, related to cattle stocking rates, has been identified as the major source of NH_3_ emissions in West African savanna regions (Delon et al. 2010). In their study, the authors developed an NH_3_ emission inventory for 23 West Africa countries, taking into account stocking rates in each region, N deposited by cattle, and NH_3_ volatilization rate of the N excreted. In general, loss rates of NH_3_ following excretion is likely in the range of 10–36% of excreted N, depending on animal category and waste management (Bouwman and Van Der Hoek 1997). Due to favorable conditions in the Sahel for NH_3_ volatilization (high temperatures, high pH, low soil moisture and often bare soils), Delon et al. (2010) applied 30% and 50% volatilization rates to N excreted, resulting in 8.4 ± 3.8 and 12.5 ± 5.9 kg N·ha^−1^·yr^−1^, respectively. Grazed savanna systems in West Africa showed rates of NH_3_ volatilization up to an order of magnitude higher compared to undisturbed savanna ecosystems without livestock, which mainly function as NH_3_ sinks (Bowden 1986). Similarly, Delon et al. (2017) observed NH_3_ volatilization rates in a Senegalese semiarid savanna close to 0, with daily NH_3_ fluxes fluctuating between −2.3 to 1.3 ng N·m^−2^·s^−1^, depending on atmospheric NH_3_ concentration, meteorology and compensation point mixing ratios.

Our calculations of N lost as NH_3_ from manure left on pasture (with 15.3% of excretion N lost as NH_3_ volatilization (IPCC 2019) and grazing time of 40%) resulted in 0.6 ± 0.2 kg N·ha^−1^·yr^−1^ (Appendix S1: Table S4). We considered mean NH_3_ emissions from soils to be negligible, following the results presented by Delon et al. (2017). Assuming that the total N excretion is 29,520 ± 8,116 kg N/a in bomas (0.6 ha), and 7,379 ± 2,029 kg N/a in piospheres (33.5 ha), and that 30% of the N excretion is lost as NH_3_ (Table 1) in bomas and 15.3% in piospheres, the NH_3_ emitted from bomas and piospheres resulted in 14,760 ± 4,058 and 34 ± 9 kg N·ha^−1^·yr^−1^, respectively. The percentage reported by the 2019 Refinement to the 2006 Guidelines for National Greenhouse gas inventories (IPCC 2019) of N loss via volatilization from bomas is around double the percentage reported of N loss from manure on pasture. This captures the positive feedback of manure accumulation on NH_3_ emissions, showing that livestock management plays a key role in the contribution of the overall N‐atmospheric budget.

#### Nitrous oxide (N2O)

In pastoral systems, soil N_2_O emissions are enhanced by the presence of animals. Urine patches are large contributors to soil N_2_O emissions in grazing systems (Gerber et al. 2014), as the urine increases N availability in the soil and, thus, the microbial production of N_2_O via nitrification and denitrification (Butterbach‐Bahl et al. 2013). Furthermore, trampling from livestock increases soil compaction and lowers soil O_2_ availability (Liu et al. 2007). This promotes anaerobic conditions and the subsequent production of N_2_O via denitrification. Thus, trampling has been found to increase soil N_2_O emissions by up to a factor of two compared to non‐compacted soils (Oenema et al. 1997). Still, the overall contribution of N_2_O emissions to the N budget in semiarid SSA savannas with low soil humidity can be considered negligible (Delon et al. 2012). Although rainfall events stimulate microbial activity, and may trigger N_2_O production through denitrification (Butterbach‐Bahl et al. 2013), experiments on biogenic N_2_O emissions from dry and wet soil often registered levels that were below the detection limit (Levine et al. 1996, Zhu et al. 2018).

Rees et al. (2006) highlighted that only a small proportion of N losses occurs via N_2_O emissions in the savanna in Zimbabwe, thereby reporting fluxes of 0.25–0.50 kg N·ha^−1^·yr^−1^. Furthermore, Scholes et al. (1997) estimated N_2_O fluxes of 0.03–0.27 kg N·ha^−1^·yr^−1^ from a South Africa savanna. Similarly, low N_2_O fluxes were reported by Brümmer et al. (2008) for a savanna in Burkina Faso, with annual means of 0.52–0.67 kg N·ha^−1^·yr^−1^, or by Butterbach‐Bahl et al. (2020) for savannas in Kenya (5.5 ± 1.0 µg N_2_O‐N·m^−2^·h^−1^ or 0.5 kg N·ha^−1^·yr^−1^). Overall, the current evidence suggests that N_2_O emissions from grazed savanna soils are quantitatively unimportant for N budgeting, although they might be of environmental concern due to the high GHG potential of N_2_O (IPCC 2013). For our virtual farm, we used a value of 0.43 ± 0.22 kg N·ha^−1^·yr^−1^, which is the mean value reported for the four available studies investigating N_2_O emissions from savanna soils. We calculated 0.008 ± 0.002 kg N·ha^−1^·yr^−1^ of N_2_O emissions from manure left in the pastures, assuming a total annual excretion of 36,900 ± 10,145 kg N/a, and that 0.2% are lost as N_2_O (IPCC 2019, Table 1). Total N_2_O emissions from pasture resulted in 0.4 ± 0.2 kg N·ha^−1^·yr^−1^ (Appendix S1: Table S4).

Considering that 0.14% of the N excreted in bomas is lost as N_2_O (Tables 1 and 3), 24.8 ± 6.8 kg N·ha^−1^·yr^−1^ of N_2_O are emitted from bomas. We calculated N_2_O emissions from piospheres by taking the percentage of N lost as N_2_O from manure left on pastures in the piospheres (0.2% of N excreted; IPCC 2019), and adding those emissions (0.4 ± 0.1) to soil N_2_O emissions (0.4 ± 0.1; Appendix S1: Table S4), resulting in 0.8 ± 0.2 kg N·ha^−1^·yr^−1^ (Appendix S1: Table S5).

**Table 3:** Annual N excretion rates in savanna, bomas, and piospheres for the dry (DS) and wet season (WS).

				Excretion (kg N/yr)			
Season	Excretion (g N·TLU^−1^·d^−1^)	Time (d)	TLU	Savanna	Bomas	Piospheres	Farms
DS	101 ± 30	240	1,858	22,519 ± 6,689	18,015 ± 5,351	4,503 ± 1,338	45,038 ± 13,378
WS	129 ± 31	120	1,858	14,381 ± 3,456	11,505 ± 2,765	2,876 ± 6,91	28,762 ± 6,911
Total	231 ± 61	360	1,858	36,900 ± 10,145	29,520 ± 8,116	7,379 ± 2,029	73,800 ± 20,289

Notes

Daily excretion rates obtained from Schlecht et al. (1995). Values are mean ± SD.

#### Dinitrogen (N2)

Fluxes of dinitrogen (N_2_) due to denitrification are only significant in water saturated soils that can hold anaerobic conditions (Wolf and Russow 2000). As most SSA savannas soils are too sandy to remain anaerobic it is plausible to assume that N_2_ emissions are low (Scholes et al. 1997) or even negligible (Delon et al. 2010). Based on modeling, Rees et al. (2006) estimated N_2_ emissions of 0.99 kg N·ha^−1^·yr^−1^ from a savanna in Zimbabwe. Scheer et al. (2020) estimated a N_2_O :  (N_2_+N_2_O) ratio value of 0.124 ± 0.031, based upon a literature review. Schlesinger (2009) estimated that the N_2_O : (N_2_+N_2_O) ratio for N gas emissions from soils under natural vegetation or recovering vegetation is ˜0.5. We used the mean of both ratios (0.4 ± 0.2 kg N·ha^−1^·yr^−1^, i.e., equal to N_2_O fluxes, and 2.8 ± 1.4 kg N·ha^−1^·yr^−1^) to estimate a value of 1.6 ± 0.8 kg N·ha^−1^·yr^−1^) for the savanna soils within our virtual farm, although this remains highly speculative. Our estimation of N_2_ fluxes from manure left on pasture, calculated as three times the N_2_O emissions from manure left on pastures (Jarvis and Pain 1994), resulted in 0.024 ± 0.006 kg N·ha^−1^·yr^−1^, resulting in 1.6 ± 0.8 kg N·ha^−1^·yr^−1^ (Appendix S1: Table S4) lost from pastures via N_2_.

Publications from other ecosystems show that a significant percentage of the N in accumulated manure is lost as N_2_. Moral et al. (2012) estimated that N_2_ and N_2_O emissions from manure were 5.2% and 1% of the initial N, respectively. However, due to the method used (jar incubation) where aeration could be altered, this value probably overestimates N_2_ losses under field conditions. Lee et al. (2011) found by N isotope fractionation that 25% of the gaseous N losses were likely in the form of N_2_. Jarvis and Pain (1994) estimated N_2_ losses from manure as 6% of the initial N in a dairy farm study on a temperate grassland (i.e., 3xN_2_O from accumulated manure). The latter value was taken to calculate N_2_ losses from the bomas in our virtual farm, yielding 2,952 ± 813 kg N·ha^−1^·yr^−1^. Calculations of N_2_ emissions from piospheres were done by adding soil emissions 0.5 ± 0.2 kg N·ha^−1^·yr^−1^ to N_2_ emitted from manure left on pasture (1.2 ± 0.3 kg N·ha^−1^·yr^−1^), resulting in 1.7 ± 0.5 kg N·ha^−1^·yr^−1^ lost from piospheres in form of N_2_ (Appendix S1: Table S5).

#### Nitric oxide (NO)

Galy‐Lacaux and Delon (2014) found that soil NO emissions accounted for 1.5–1.6 kg N·ha^−1^·yr^−1^ for three sites in a West African savanna, or 17% of the total N gas emissions, (excluding N_2_). Similar magnitudes of soil NO emissions were obtained by modeling (1.4 ± 0.3 kg N·ha^−1^·yr^−1^) (Delon et al. 2012). Comparable annual NO fluxes of ˜1.6 kg N·ha^−1^·yr^−1^, with average NO fluxes of 0.4 (0.3–1.5) kg N·ha^−1^·yr^−1^ and 2.5 (0.4–0.44) kg N·ha^−1^·yr^−1^ in dry and wet seasons respectively, were also simulated in a savanna in South Africa (Otter et al. 1999). These values were in the same range as the annual NO fluxes (1.5 kg N·ha^−1^·yr^−1^) measured by (Scholes et al. 1997). Levine et al. (1996) reported a range of 0.1–2.0 kg N·ha^−1^·yr^−1^ for dry season and 1.8–10.7 kg N·ha^−1^·yr^−1^ for the wet season in Kruger National Park in South Africa. Meixner et al. (1997) measured soil NO emissions from a savanna site in Zimbabwe of around 0.1 kg N·ha^−1^·yr^−1^ in the dry season and 1.4 kg N·ha^−1^·yr^−1^ in the wet season. This suggests that NO fluxes in the wet season might be elevated by one order of magnitude compared to the dry season. All NO flux results from studies in semiarid African savannas were in the same range (Appendix S1: Table S4), with flux estimates varying with soil moisture content, <0.5 kg N·ha^−1^·yr^−1^ during the dry season and large pulses (e.g., 300 µg N·m^−2^·h^−1^) after rain events caused by a dormant water‐stressed microbial community in dry savanna soils (Brümmer et al. 2008). For our study, we averaged values from studies reporting soil NO fluxes for dry (0.5 ± 0.1 kg N·ha^−1^·yr^−1^) and wet (2.2 ± 0.4 kg N·ha^−1^·yr^−1^) seasons, resulting in annual soil NO emission of 1.1 ± 0.2 kg N·ha^−1^·yr^−1^, although we predict that the range could be 0.1 to ∼11 kg N·ha^−1^·yr^−1^.

#### Hydrological nitrogen losses

Hydrological N losses in savanna occur through surface runoff and leaching from soils or from manure left on pastures by grazers (IPCC 2019). Environmental conditions, including rainfall patterns, have been shown to affect N dynamics in pastoral systems across arid and semiarid savannas in SSA (Huntley 1982, Lulla 1987), with ${\text{NO}}_{3}^{‐}$ leaching, which dominates the overall N leaching, and dissolved organic nitrogen (DON), usually confined to wet seasons, as only during this period is significant rainfall observed. In East African savannas, rainfall follows a bimodal pattern, whereas Southern and West African savannas are characterized by unimodal rainfall patterns with a short wet season lasting from October to January in Southern Africa (Meixner et al. 1997) and from June to September in West Africa (Schlecht et al. 2004).

Leaching may be higher in our virtual farm, under bimodal rainfall patterns, than in other regions in SSA, with only one rainy season. Furthermore, livestock urine has a high impact on ${\text{NO}}_{3}^{‐}$ leaching in pastoral systems (Di and Cameron 2002), as high peaks of ${\text{NO}}_{3}^{‐}$ leaching might occur under urine patches (Silva et al. 1999). While information on ${\text{NO}}_{3}^{‐}$ leaching is relatively abundant for agricultural soils worldwide (e.g., Smaling et al. 1993, Ledgard 2001), for SSA pastoral systems we found one study investigating ${\text{NO}}_{3}^{‐}$ leaching, in a semiarid savanna in Zimbabwe (Rees et al. 2006), which reported losses of 2–3 kg N·ha^−1^·yr^−1^. We could not find studies reporting values specifically for N losses from urine patches or through erosion in semiarid regions in SSA, although Robertson and Rosswall (1986) estimated the combination of erosion, runoff and leaching in West Africa to result in N losses of 5.1 kg N·ha^−1^·yr^−1^. Similarly, DON is known to contribute substantially to N leaching in forests, but very little is known of its contribution in agricultural soils (Siemens and Kaupejohann 2002). While we could not find studies reporting on DON leaching from savanna soils, we found evidence that DON is an important contributor to N leaching in grasslands soils with N urine input, and therefore must be considered in N cycling studies. Several authors studied ${\text{NO}}_{3}^{‐}$ and DON leaching from grassland soils, including urine and dung patches (a global grasslands review [Cai and Akiyama 2016] and two field studies in grasslands of Germany [Wachendorf et al. 2005, Siemens and Kaupejohann 2002]). The three studies reported N lost through leaching to be around 30% as DON and 70% in form of ${\text{NO}}_{3}^{‐}$ from both soils and manure, with very little or nothing lost as NH_4_^+^ (Wachendorf et al. 2005), values used for our calculations.

For our virtual farm we took 2.5 ± 0.5 kg N·ha^−1^·yr^−1^ as N lost from soils through ${\text{NO}}_{3}^{‐}$ leaching (Rees et al. 2006). We added 0.1 ± 0.0 (3.5% of excreted N) from manure left on pastures, assuming a total of 2.6 ± 0.5 kg N·ha^−1^·yr^−1^ lost as ${\text{NO}}_{3}^{‐}$ leaching. For DON losses from savanna soils, we calculated 1.1 ± 0.2 kg N·ha^−1^·yr^−1^ as 30% of total N leached, considering that N lost as ${\text{NO}}_{3}^{‐}$ (2.6 ± 0.5 kg N·ha^−1^·yr^−1^) represented 70% of total N leached.

Data on leaching losses from manure management systems in SSA are extremely limited. We assumed that 3.5% of N excreted is lost through leaching in dry environments (IPCC 2019) in ${\text{NO}}_{3}^{‐}$ losses from bomas of 1,732 ± 473 kg N·ha^−1^·yr^−1^ and 10.2 ± 8.2 kg N·ha^−1^·yr^−1^ from piospheres for our virtual farm (Appendix S1: Table S5). If we consider these values as 70% of total N leached as ${\text{NO}}_{3}^{‐}$, we estimated 742 ± 203 kg N·ha^−1^·yr^−1^ and 4.4 ± 3.5 kg N·ha^−1^·yr^−1^ lost as DON from bomas and piospheres, respectively.

#### Nitrogen in animal products

One of the main functions of raising livestock in pastoral systems in SSA is to provide products (milk, meat, leather) for subsistence and for generating incomes when sold in local markets (Otte and Chilonda 2002). When these products are consumed or sold, N is exported out of the system. Annual meat and beef offtake in traditional systems is extremely low compared to other livestock systems (Otte and Chilonda 2002). The annual milk offtake (milk that is not drunk by the calf) estimated for cattle in arid and semiarid pastoral areas is 58 kg·TLU^−1^·yr^−1^ (Otte and Chilonda 2002). There has been very little research in N partitioning into milk for African cattle breeds, especially for pastoral systems. We assumed an N partition weight ratio of 5 g N/kg milk, based on (Rufino et al. 2006) for dairy cows in a mixed farming system in Africa. Thus, we estimated that N losses due to milk export are 1.4 g N·TLU^−1^·d^−1^ during the lactation period (i.e., 210 d), or 0.3 kg N·TLU^−1^·yr^−1^. Considering a cattle density of 0.19 TLU/ha for our virtual farm (Table 1), losses of N via export of milk resulted in 0.06 kg N·ha^−1^·yr^−1^. This very low value was expected due to the extremely low milk production and cattle density in pastoral systems compared to other livestock systems in SSA (e.g., 0.35–0.55 TLU/ha in smallholders in East Africa; Rufino et al. 2006).

Estimates of animal offtake rates for pastoral herds are between 6–15% of the total heard, depending on the district, season, and year (Farmer and Mbwika 2012). We took an intermediate value of ˜10% to estimate the number of animals sold, which applied to our virtual farm resulted in 186 TLU (10% of a herd of 1,858 TLU). Considering that each animal´s meat offtake in arid and semiarid pastoral systems is 16.5 kg/TLU (Otte and Chilonda 2002), our offtake estimation is ˜3,000 kg/yr of meat. Meat contains between 10% and 15% of protein (Syrstad 1993), and given an N content in proteins of 16% (N × 6.25; Satter and Roffler 1975), the total annual N export via meat is estimated to be 48–72 kg N/yr (or 0.004–0.007 kg N·ha^−1^·yr^−1^) from our virtual farm.

### N balance in pastoral systems in SSA

**Fig. 1:**
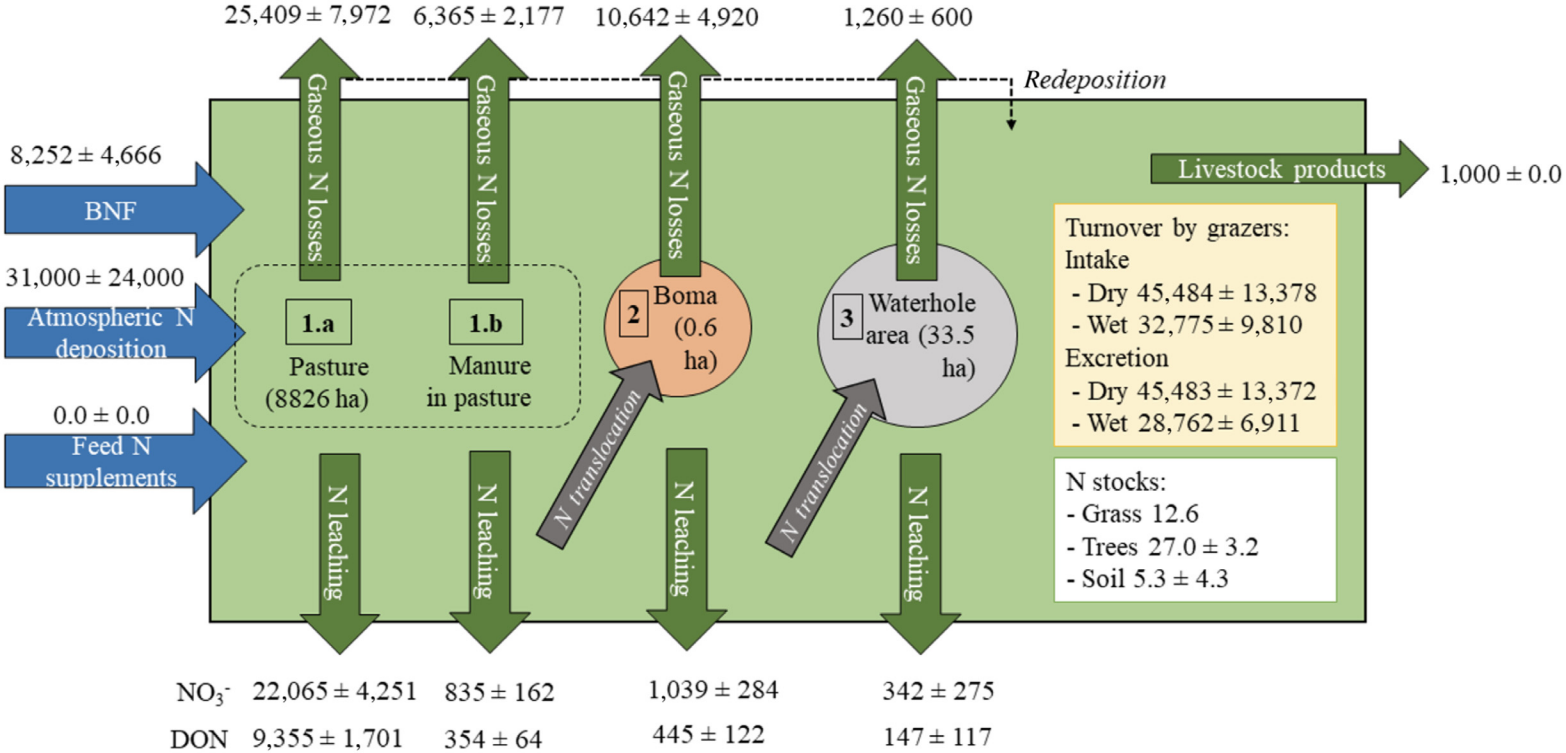
Nitrogen flows (kg N/yr) and stocks (kg N/ha) calculated for a virtual farm of 10,000 ha. Simplified scheme (not to scale) showing N dynamics in pastoral systems in sub‐Saharan Africa. The system area is represented in light green. The area comprises the pasture (1.a) and the urine/feces therein (1.b, in dark brown), the bomas (2, in light brown) and the piosphere (3, in light blue). N flows are represented with arrows (N inputs blue arrows; N outputs red arrows; internal N translocations with green arrows, e.g., from the pasture (1.a) to congregation areas. Some of the NH_3_ can be redeposited into the system (dotted arrow). Vegetation and soil N stocks (white box) are included in the budget, and turnover of N by grazers (i.e., intake and excretion, yellow box) are used for N fluxes calculations. BNF stands for biological nitrogen fixation.

Nitrogen fluxes from Appendix S1: Table S4 and Appendix S1: Table S5 were extrapolated to their respective source areas to quantify the contribution of each N flux to total N inputs and losses in our virtual farm (Table 4). N fluxes (kg N·ha^−1^·yr^−1^) estimated from publications were multiplied by the area of the farm where these fluxes occur (Table 4), resulting in the annual N input and output (kg N/a) for each area (Fig. 1), and its contribution to the total N budget (%). Piospheres accounted for a total area of 33.5 ha (6.7 ha/waterhole × 5 waterholes), bomas covered only 0.6 ha (0.03 ha/boma × 19 bomas) (Table 1), while savanna soils occupied 8,826 ha. A further 1,120 and 20 ha are accounted for by exposed soil and standing water, respectively (Appendix S1: Table S3).

**Table 4:** Summary table of N fluxes from each source, source area, farm N inputs, and outputs and contribution to the total N fluxes (%).

Source	N flux values (kg N·ha^−1^·yr^−1^)	Area (ha)	N inputs (kg N/yr)	N outputs (kg N/a)	Contribution to total (%)	
					N input	N output
BNF	7.4 ± 4.3†	900	8,252 ± 4,666		21	
BNF	0.2 ± 0.1†	7,960				
N atmospheric deposition	3.1 ± 2.4†	10,000	31,000 ± 24,000		79	
N feed supplements‡	0 ± 0	10,000	0 ± 0		0	
NH_3_ savanna	0.5 ± 0.2	8,826		4,413 ± 2,206		6
N_2_O savanna	0.4 ± 0.2	8,826		3,530 ± 1,765		4
N_2_ savanna	1.6 ± 0.8	8,826		14,122 ± 7,061		18
NO savanna	1.1 ± 0.2	8,826		9,709 ± 1,765		12
NO_3_ leaching savanna	2.6 ± 0.5	8,826		22,900 ± 4,413		29
DON leaching savanna	1.1 ± 0.2	8,826		9,709 ± 1,765		12
Animal products offtake	0.1 ± 0	10,000		1,000 ± 0		1
NH_3_ bomas	14,760 ± 73,80	0.6		8,856 ± 4,428		11
N_2_O bomas	24.8 ± 6.8	0.6		15 ± 4		<1
N_2_ bomas	2,952 ± 813	0.6		1,771 ± 488		2
NO_3_ leaching bomas	1,732 ± 473	0.6		1,039 ± 284		1
DON leaching bomas	742 ± 203	0.6		445 ± 122		1
NH_3_ piospheres	34 ± 17	33.5		1,139 ± 569		1
N_2_O piospheres	0.8 ± 0.2	33.5		27 ± 7		<1
N_2_ piospheres	1.7 ± 0.5	33.5		57 ± 17		<1
NO piospheres	1.1 ± 0.2	33.5		37 ± 7		<1
NO_3_ leaching piospheres	10.2 ± 8.2	33.5		342 ± 275		<1
DON leaching piospheres	4.4 ± 3.5	33.5		147 ± 117		<1
Total N input/output			39,252 ± 28,666	79,258 ± 25,293		100
N balance (kg N/a)	−40,006 ± 3,373					
N balance (kg N·ha^−1^·yr^−1^)	−4 ± 1					

Notes

Flux, input, and output values are mean ± SD. BNF, biological nitrogen fixation; DON, dissolved organic nitrogen.

† Error estimates based on a single publication.

‡ Feeding supplements to livestock is not a common practice in traditional livestock systems in SSA.

Calculations of the N balance for our virtual farm resulted in moderate loss close to neutral (i.e., −4 ± 1 kg N·ha^−1^·yr^−1^). Major N losses resulted through ${\text{NO}}_{3}^{‐}$ leaching, followed by NO and N_2_ fluxes in the savanna and NH_3_ in the bomas. Around 16% of the total N losses from the system occurred in the bomas, even if they contribute <0.01% of the land area.

The N balance for our virtual farm in an East African savanna is closer to zero than the N budget calculated for a Cerrado savanna in Brazil (16–44 kg N·ha^−1^·yr^−1^). The Cerrado savanna is also dominated by N input through BNF, though with a higher canopy cover (50%) than our virtual farm in Kenya (9%). However, the N balance across SSA is expected to be different in fertile savannas, with a higher N‐fixing stand density (resulting in higher BNF rates) and in areas with different management practices (e.g., higher grazing rates) or more frequent fire events. N inputs in our virtual farm through BNF were estimated to be 21% of the N inputs, whereas atmospheric N deposition accounted for 79%. Major N losses from the system occur via N leaching from savanna soils, contributing to 41% (29% ${\text{NO}}_{3}^{‐}$ and 12% DON) of the N losses from the system, followed by N_2_ emissions from pasture (18%), biogenic NO emissions from pasture (12%) and NH_3_ volatilization from bomas (11%).

Most N balances for livestock systems in SSA in the literature (i.e., mixed cropped systems/agro‐pastoral systems) are negative (Cobo et al. 2010), i.e., indicating net losses of N from the system. Results from this study suggest that the net N budget from our virtual farm is close to zero, though a significant amount of N is translocated within the system. All the N found in the bomas comes from pastures, via intake and excretion, leading to N depletion in pastures. N losses in the systems increase if NH_3_ volatilization from wildlife manure and emissions of N‐compounds from biomass burning are taken into account. Assuming a density of 800 wildebeest/km^2^ per day results in N losses via NH_3_ volatilization of 2 kg N·ha^−1^·yr^−1^ (Ruess and McNaughton 1988). Biomass burning frequency varies from approximately 3 yr in infertile savannas to 5 yr in fertile savannas in regions with 600 mm mean annual precipitation (Scholes et al. 1996). Direct NO_x_ losses from fires in dry savannas, which are linearly dependent on the N content of the fuel (Lacaux et al. 1996), are reported to be 1.5 ± 0.3 kg N·ha^−1^·yr^−1^ in West Africa (Galy‐Lacaux and Delon 2014), 4.4–5.1 g/kg DM (Scholes et al. 1996) and 1.5–2 g N (NO_x_)/kg DM (Lacaux et al. 1996) in southern African savannas. Considering that our estimations of DM are 2,980 kg/ha, we estimate that 4,470–15,198 kg N/ha could be lost from biomass burning in our virtual farm, showing that when fires take place, it can become the major pathway of N loss, nevertheless several studies show that soil N levels in African savanna are not significantly affected by annual burning over long periods of time (Moore 1960, Trapnell et al. 1976, Coetsee et al. 2008). Considering N lost from biomass burning, our N balance for the virtual farm changes by approximately 15–50%.

It is noteworthy that other factors substantially change N dynamics on pastoral systems, such as livestock species. In some regions of SSA, goats are the main form of livestock, often as a strategy for survival in environments where rainfall and feed resources are unpredictable (Mace and Houston 1988). Cattle need productive pasture, which together with the animals themselves, are competitive resources. This can result in armed conflicts and cattle raids (Österle 2008). In contrast, goats are browsers and unlike cattle, they are able to feed on the bushy vegetation and benefit from bush encroachment. This causes changes in the sub‐canopy tree dynamics, and therefore patterns of vegetation distribution affecting tree–grass interaction in savannas (Scholes and Archer 1997), and ultimately the N cycle.

### Knowledge gaps on N cycling in pastoral systems of SSA

The level of evidence of N flows and their magnitudes involved in the N cycle of pastoral systems in semiarid SSA is low (Table 5). The major gaps in knowledge on N cycling in the pastoral systems of SSA were found to be the uncertainty (e.g., 100% uncertainty) of key N fluxes associated to N flux calculations, such as N lost as NH_3_ volatilization or ${\text{NO}}_{3}^{‐}$ leaching from manure. We also found that livestock‐related N flows were often excluded from N balance calculations. Bomas and waterholes have rarely been studied with regard to their importance as hotspots for N losses. N fluxes that present difficulties to be measured, such as N_2_ emissions and DON leaching from soils and manure, are excluded from all N balance studies in SSA pastoral systems despite the fact that our study indicates that the amount of N lost in the form of N_2_ and DON may be high. Furthermore, studies often do not cover spatial (e.g., tree density, hotspots) and temporal variations (i.e., dry/wet seasons, interannual variabilities). The N budget at a system scale in this study resulted slightly negative (net loss of N), for a relatively nutrient‐poor savanna, although savanna N dynamics in other regions of SSA may vary, with soil fertility as the main driver.

**Table 5:** Rating of N flows importance, measurement complexity, evidence availability, and assessment of uncertainty.

N flows	Potential importance for N budget calculation	Technical complexity to measure	Evidence available	Overall assessment of uncertainty
BNF	High	High	Low	High
Atmospheric deposition	Medium	Medium	Medium	Low
N supplements	Low	Low	NA	NA
NH_3_ from congregation areas	Medium	High	Low	High
N_2_O from congregation areas	Medium	Medium	Low	High
N_2_ from congregation areas	Medium	High	Low	High
N leaching (${\text{NO}}_{3}^{‐}$ and DON)	High	Low	Low	Medium
NH_3_ from savanna soils	Low	High	Low	Medium
N_2_O from savanna soils	Low	Medium	Low	Medium
N_2_ from savanna	Low	High	Low	High

Note

NA, not available.

### Practical recommendations for managers

Management strategies and proper decision‐making processes, such as planning, acting, and monitoring are key to successful livestock systems (Cross et al. 2004). First, the identification of major N input and output processes in semiarid pastoral systems through simplified N balances as monitoring and planning tools, can provide a basis for nutrient management assessment (Lanyon and Beegle 1989). Secondly, strategies such as anticipating the animal stock that an area is able to support, and planned rotational grazing that allows vegetation to recover, are key management actions for sustainable use and restoration of pasture (Teague and Dowhower 2003). Lastly, reducing accumulation of manure in bomas as a strategy to reduce N losses and compaction, by increasing mobility of the enclosures and reducing the enclosure period to 2–3 d is another valuable option for better farm management (Harris 2002).

## Conclusions

This review has developed for the first time a full N balance for a pastoral system in SSA. Several relevant N flows in these LPS have not been investigated before, such as gaseous N losses from congregation areas or N leaching and will need new data collection. Uncertainty in major N fluxes in semiarid regions, such as NH_3_ volatilization rate and N leaching from manure remains high (50–100%), causing a high variation of the N budget. The change of BNF rates depending upon the intertwined relationship between N‐fixing tree density and soil fertility, has not been well studied, resulting in an N flow that can drastically alter the final N balance. Along with the ongoing intensification of these LPS in SSA, stocking rates are likely increasing. More accurate livestock census data in pastoral systems in SSA are needed as a background information to assess changes in stocking rates, as a prerequisite to evaluate to which extent livestock changes and herd mobility affect the N balance. According to the limited information, enhancements of stocking rates might cause larger NH_3_ emissions and ${\text{NO}}_{3}^{‐}$ leaching, depending on cattle feeding quality and quantity, as well as climatic conditions and soil texture. Therefore, this will potentially lead to different intensities of eutrophication of terrestrial and aquatic ecosystems and acidification of SSA savanna soils. Based on the knowns and unknowns highlighted in this review, researchers are enabled to aim future research towards closing knowledge gaps in N cycling in sub‐Saharan African pastoral systems. We highlight the urgent need of collecting experimental data on BNF and N_2_ emissions, gaseous losses from bomas and N leaching losses, to improve the N balance estimations as indicators of land degradation for further management assessment.

Findings in this review prove that many different factors in savanna ecosystems affect the final N budget. Understanding the underlying processes between climate, soils, animals, and humans and identifying major N input and output pathways, will lead to appropriate management practices that will help avoiding potential irreversible degradation of these valuable ecosystems. Particularly the importance of animal enclosures and thus avenues for improved management to recycle valuable nutrients beyond N needs further investigation.

## Supporting information

Appendix S1

## Footnotes

^8^

http://www.isric.org

^9^

http://www.fao.org/geonetwork/

^10^

https://www.sentinel‐hub.com/
