# Supplementary material for: Nitrogen cycling in pastoral livestock systems in Sub‐Saharan Africa: knowns and unknowns
Source: Ecol Appl. 2021 Jun 10;31(6):e02368. doi: 10.1002/eap.2368 (PMC8459270; doi:10.1002/eap.2368)
Supplement: Supplementary file 1 — Appendix S1 [file EAP-31-e02368-s001.pdf]

Supporting Information. Carbonell, V., L. Merbold, E. Díaz-Pinés, T. Dowling, and K. Butterbach-Bahl. 2021. Nitrogen cycling in pastoral livestock systems in Sub-Saharan Africa: knowns and unknowns. Ecological Applications.

Appendix S1. Additional tables and figures mentioned in the main text.

Table S1. Keywords used in literature search. Organized by main keywords and N flow/stock specific keywords. Number of results for each category are indicated in parenthesis.

Main keywords	N inputs and outputs/export	Soil and vegetation nitrogen stocks	N emissions from bomas and waterholes
Nitrogen AND savanna AND Africa (369)	BNF (17)	Soil N stocks (58)	Ammonia manure (3)
	Deposition (77)	Vegetation N stocks (34)	Nitrous oxide manure (1)
	Nitrous oxide (56)	Biomass (25)	N_2 manure (5)
	Ammonia (65)		Bomas (6)
	Leaching (74)		Kraals (11)
	Nitric oxide (56)		Enclosures (7)
	N_2 (99)		Watering point (21)

Table S2. Mean ± standard deviation (sd) N stocks (Mg N ha^{-1}) by soil type for 0-100 cm depth.

	Soil type*	# profiles	0-100 cm
N stocks	Cambisols	4	6.2±2.1
(Mg N ha^{-1})	Ferrasols	2	6.5±0.5
	Gleysols	16	5.6±2.5
	Luvisols	15	6.8±4.6
	Arenosols	68	4.9±4.0
	Regosols	53	4.3±3.3
	Vertisols	20	5.3±2.3
	all soils	178	5.0±3.6

*(IUSS Working Group WRB, 2014)

Table S3. Summary table of land cover percentage (%) in a pastoral farm in semiarid Kenya.

Class	Land cover (%)
Canopy vegetation (%)	9.0
Other vegetation (%)	79.6
Surface water (%)	0.2
Exposed soil (%)	11.2

Values derived from random forest classification on Sentinel 2 imagery. Rand accuracy=0.97, indicating that 97% of test pixels are classified correctly using the model found by the evaluation of the training pixels.

Table S4. Nitrogen fluxes from pasture in pastoral systems in sub-Saharan Africa.

Flux	N flux (kg N ha^{-1} a^{-1})	Region	Source	Method
BNF	16.4, 30.2 and 44[a]	Global tropical savanna ecosystem	Cleveland et al. (1999)	Calculated as the average of values from literature
	18.6	African savanna	Chen et al. (2010)	Estimated from Cleveland et al. (1999) corrected according to Fischer et al. (2008)
	20-60	Tropical savanna ecosystem	Houlton et al. (2008)	Applied model to data from literature
	30	Savanna in West Africa	Robertson and Roswall (1986)	Sum of symbiotic, algal and other BNF values from publications
	**7.4** ± **4.3** (trees), **0.2** ± **0.1** (herbs)	*Tanzanian savanna /virtual farm in Kenyan savanna*	Cech et al. (2010)	Measured biomass data and tissue N concentration for trees and herbs
Atmospheric N deposition	7.4 (1.0)	Savanna in West Africa	Delon et al. (2012)	Surface measurements, satellite imagery and modelling wet and dry deposition
	6 (1.8)	Savanna in West Africa	Galy-Lacaux and Delon (2014)	Data from long-term measurements from 10 IDAF project stations.
	**3.1** ± **2.4**	*virtual farm in Kenyan savanna*	Dentener (2006)	Values in pastoral regions in SSA extracted from global data set generated using a model for wet and dry deposition
Ammonia (NH_3) emissions	-0.44	Savanna in Senegal	Delon et al. (2017)	Field observations (dynamic Teflon chambers)
	8.4 ± 3.8, 12.5 ± 5.9[b]	Savanna in West Africa	Delon et al. (2010)	Calculated from Schlecht et al (1995) data on livestock N excretion and applying 30% and 50% vol.rate
	**0.6** ± **0.2**	*virtual farm in Kenyan savanna*	Schlecht et al. (1995); IPCC (2019) *	Calculated from Schlecht et al (1995) data on livestock N excretion and applying 15.3% vol.rate
Nitrous oxide (N_2O) emissions	0.25 - 0.5	Savanna in Zimbabwe	Rees et al. (2006)	Static greenhouse gas chambers and denitrification-decomposition model (DNDC)
	0.03 – 0.27	Savanna in South Africa	Scholes et al. (1997)	manual GHG chambers
	0.52, 0.67	Savanna in Burkina Faso	Brümmer et al. (2008)	manual GHG chambers
	**0.4** ± **0.2**[c]	*virtual farm in Kenyan savanna*	Rees et al. (2006); Scholes et al. (1997); IPCC(2019)	Mean value of the three studies plus manure left on pastures*
Dinitrogen (N_2) emissions	0.99	Savanna in Zimbabwe	Rees et al. (2006)	Modelled (using DNDC) and measured data
	**0.5** ± **0.2**[d]	*virtual farm in Kenyan savanna*	Schlesinger (2009); Jarvis and Pain (1994);	No information for savanna systems. We used general assumptions as outlined in two publications: soil N_2O emission (Schlesinger, 2009) and 3x manure N_2O emissions (Jarvis and Pain, 1994)

	1.5-1.6	Savanna in West Africa	Galy-Lacaux and Delon (2014)	ISBA (Interactions between Soil, Biosphere, and Atmosphere) model
	1.4 ± 0.3	Savanna in West Africa	Delon et al. (2012)	ISBA model
	1.5	Savanna in South Africa	Otter et al. (1999)	Calculated from field observations and the Hot Wet model by Scholes and Carter (Personal communication, 1998)
Nitric oxide (NO) emissions	1.5	Savanna in South Africa	Scholes et al. (1997)	manual GHG chambers
	0.1 - 2.0[e]; 1.8 - 10.7[f]	Savanna in South Africa	Levine et al. (1996)	manual GHG chambers
	0.1[e]; 1.4[f]	Savanna in Zimbabwe	Meixner et al. (1997b)	manual GHG chambers
	**0.5 ± 0.1[e]; 2.2 ± 0.4[f]**	*virtual farm in Kenyan savanna*	Meixner et al. (1997b); Levine et al. (1996); Otter et al. (1999)*	Average for dry and wet seasons from literature
	2-3	Savanna in Zimbabwe	Rees et al. (2006)	Field observations using static and closed plastic chambers
NO_3^- leaching	5.1[g]	Savanna in West Africa	(Robertson and Rosswall 1986)	Estimated from literature values based on Niger River fluxes
	**2.6 ± 0.5[h]**	*virtual farm in Kenyan savanna*	Rees et al. (2006); IPCC (2019)	Mean value from Rees et al. (2006) plus N leached from manure left on pasture as 3.5% of N excreted
DON leaching	**1.1±0.2**	*Grasslands / virtual farm in Kenyan savanna*	Siemens and Kaupejohann 2002; Wachendorf et al. 2005; Cai and Akiyama 2016	Calculated as 30 % of total N leached, from NO_3^- leached as the remaining 70 %
N milk and meat offtake	**0.1**	*virtual farm in Kenyan savanna*	Otte and Chilonda(2002); Rufino et al. (2006); Farmer and Mbwika(2012); Syrstad (1993); Satter and Roffler (1975)*	Calculated from published values, with milk and meat offtake, N and protein partitioning and cattle density

[a] estimations for 5%, 15% and 25% of tree cover

[b] NH_3 volatilization rates of 30% and 50% of added/deposited N

[c] Result from adding our calculations of N_2O emitted from manure left on pasture (0.008±0.002 kg N ha^{-1} a^{-1}) to mean value from literature for N_2O emissions from savanna soils (0.43±0.22 kg N ha^{-1} a^{-1})

[d] N_2O:(N_2+N_2O) ratio of 0.5. Result from adding N_2 emitted from pastures (0.43±0.22 kg N ha^{-1} a^{-1}) to N_2 emitted from manure left on pasture (0.024±0.0006 kg N ha^{-1} a^{-1})

[e] dry season

[f] wet season

[g] Includes N lost as leaching, runoff and erosion

[h] Result from adding N leached from pasture (2.5±0.5 kg N ha^{-1} a^{-1}) (Rees et al, 2006) to N leached from manure left on pasture (0.1±0.0 kg N ha^{-1} a^{-1}) (IPCC (2019).

* Calculated from excretion rates for dry / wet seasons (Table 7)

Table S5. Nitrogen fluxes (mean ± sd) in bomas and piospheres for the virtual farm in Kenya (10000 ha). All values in kg N ha^{-1} a^{-1}, calculated from excretion rates for dry and wet season respectively (Table 7)

Flux	N flux value	Source	Method
Ammonia (NH_3) volatilization from bomas	14760±7380[a]	Schlecht et al. (1995); Delon et al. (2010); IPCC (2006)	Calculated from Schlecht et al (1998) data on livestock N excretion and applying 30% vol.rate
Nitrous oxide (N_2O) emissions from bomas	24.8±6.8	Schlecht et al. (1995); Zhu et al. (2019)	Calculated from Schlecht et al (1998) data on livestock N excretion and applying 0.14% and vol.rate
Dinitrogen (N_2) emissions from bomas	2952 ± 813	Jarvis and Pain (1994)	Calculated as 6% of excretion or three times higher than N_2O emissions
NO_3^- leaching from bomas	1732 ± 473	IPCC (2019)	3.5% of N excreted
DON leaching from bomas	742±203	Siemens and Kaupejohann 2002; Wachendorf et al. 2005; Cai and Akiyama 2016	Calculated as 30 % of total N leached, from NO_3^- leached as the remaining 70 %
Ammonia (NH_3) volatilization from piospheres	34 ± 17	Schlecht et al. (1995); IPCC (2019)	Calculated from Schlecht et al (1998) data on livestock N excretion and applying 15.3% vol.rate
Nitrous oxide (N_2O) emissions from piospheres	0.8 ± 0.2[b]	Schlecht et al. (1995); IPCC (2019)	Calculated from Schlecht et al (1998) data on livestock N excretion and applying 0.2% of N excreted plus N_2O from soils
Dinitrogen (N_2) emissions from piospheres	1.7 ± 0.5[c]	Schlesinger (2009); Jarvis and Pain (1994)	Same N_2O value from soils and three times higher than N_2O from manure left on pasture
Nitric oxide (NO) emissions from piospheres	1.1 ± 0.2	Meixner et al. (1997b); Levine et al. (1996); Otter et al. (1999)	NO emissions rate from soils (Table 6)
NO_3^- leaching from piospheres	10.2 ± 8.2[d]	IPCC (2019)	Calculated as 3.5% of N excreted plus leaching from pasture
DON leaching from piospheres	4.4±3.5	Siemens and Kaupejohann 2002; Wachendorf et al. 2005; Cai and Akiyama 2016	Calculated as 30 % of total N leached, from NO_3^- leached as the remaining 70 %

[a] IPCC 2006 Guidelines for national Greenhouse gas inventories and the 2019 refinement to the 2006 IPCC report values for nitrogen loss due volatilization of NH_3 and NOx, assuming that most N volatilized (~93%) occurs in form of NH_3, the rest 7% is estimated to occur as NOx.

[b] Result from adding our calculations of N_2O emitted from manure left on pasture (piospheres soil) (0.4±0.1 kg N ha^{-1} a^{-1}) to mean value from literature for N_2O emissions from savanna soils (0.43±0.22 kg N ha^{-1} a^{-1})

[c] Result from adding N_2 emitted from soils (0.5±0.2 kg N ha^{-1} a^{-1}) to N_2 emitted from manure left on pasture (1.2±0.3 kg N ha^{-1} a^{-1})

[d] Result from adding N leached from pasture (piospheres soil) (2.5±0.5 kg N ha^{-1} a^{-1}) to N leached from manure left on piospheres (7.7±7.7 kg N ha^{-1} a^{-1})

Figure S1. Flow diagram depicting the methodology followed in this study.

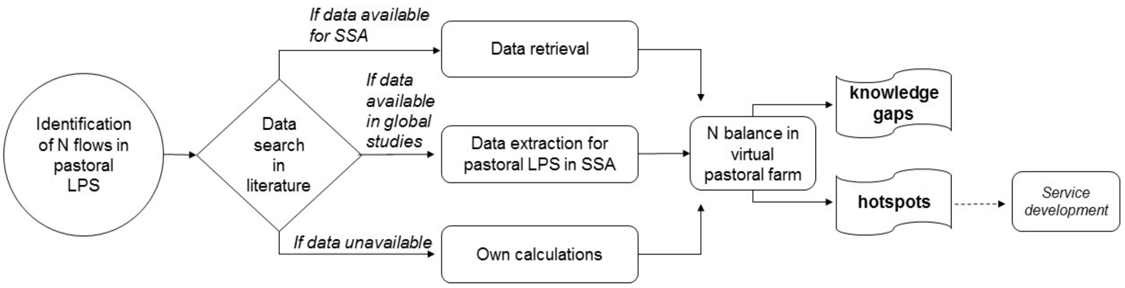

Figure S2. Geographic distribution of livestock only rangeland based (LGA) systems in arid/semi-arid sub-Saharan Africa (Seré and Steinfeld 1996) in yellow. Soil profiles selected from the Africa soil profiles database (Leenaars 2013) are represented by pink dots, and Kapiti Research Station in Kenya is shown in green. Base map source: FAO GeoNetwork (http://www.fao.org/geonetwork/)

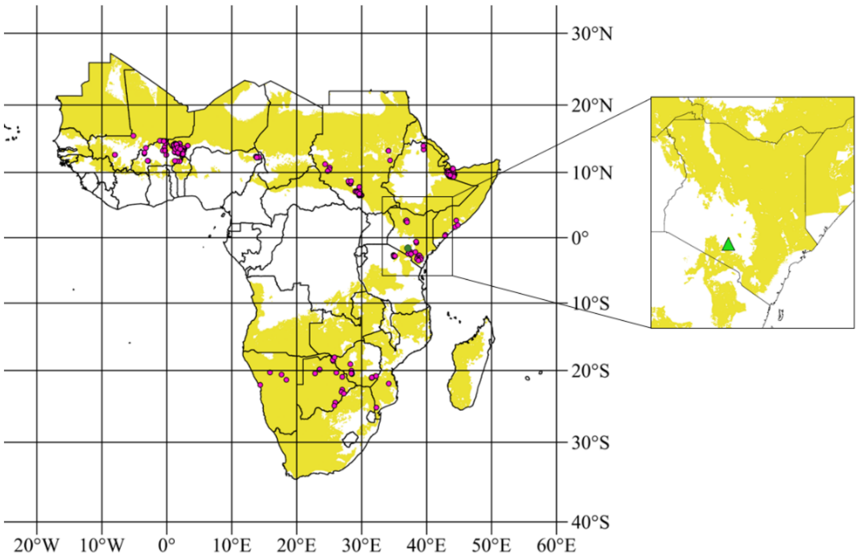

Figure S3. Procedure implemented for estimating N_2O and NH_3 losses from cattle excretions ((Delon et al. 2010). [a]N excreted in dry or wet seasons, [b] 50% grazing, 40% in bomas and 10% around waterholes, [c] volatilization rates for N_2O and NH_3

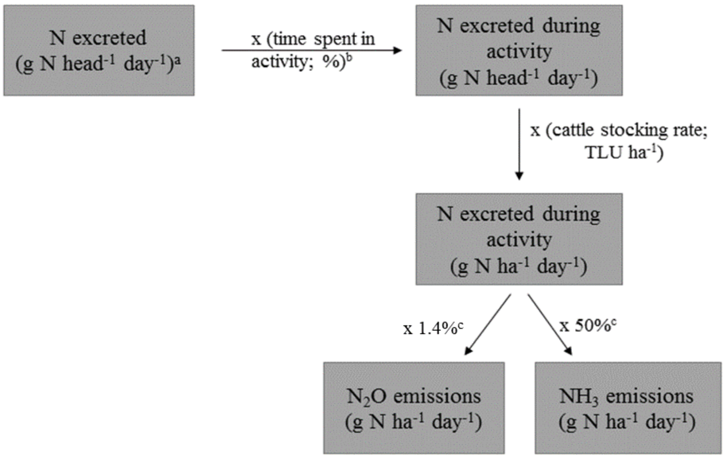

References

Andrew, M. H. 1988. Grazing Impact in Relation Watering Points Livestock. Trends in Ecology & Evolution **3:**336–339.

Brümmer, C., N. Brüggemann, K. Butterbach-Bahl, U. Falk, J. Szarzynski, K. Vielhauer, R. Wassmann, and H. Papen. 2008. Soil-atmosphere exchange of N_2O and NO in near-natural savanna and agricultural land in Burkina Faso (W. Africa). Ecosystems **11:**582–600.

Cai Y., H. Akiyama. 2016. Nitrogen loss factors of trace gas emissions and leaching from excreta patches in grassland ecoystems: A summary of available data. Science of the Total Environment **572:**185-195.

Chen, Y., J. T. Randerson, G. R. Van der Werf, D. C. Morton, M. Mu, and P. S. Kasibhatlas. 2010. Nitrogen deposition in tropical forests from savanna and deforestation fires. Global Change Biology **16:**2024–2038.

Cleveland, C. C., A. R. Townsend, D. S. Schimel, H. Fisher, L. O. Hedin, S. Perakis, E. F. Latty, C. Von Fischer, A. Elseroad, and M. F. Wasson. 1999. Global patterns of terrestrial biological nitrogen (N_2) fixation in natural ecosystems. Global Biogeochemical Cycles **13:**623–645.

Davidson, E. A. 2009. The contribution of manure and fertilizer nitrogen to atmospheric nitrous oxide since 1860. Nature Geoscience **2:**659–662.

Delon, C., M. Adon, C. Liousse, and D. Serc. 2012. Nitrogen compounds emission and deposition in West African ecosystems : comparison between wet and dry savanna. Biogeosciences **9:**385–402.

Delon, C., C. Galy-Lacaux, A. Boone, C. Liousse, D. Serça, M. Adon, B. Diop, A. Akpo, F. Lavenu, E. Mougin, and F. Timouk. 2010. Atmospheric nitrogen budget in Sahelian dry savannas. Atmospheric Chemistry and Physics Discussions **9:**14189–14233.

Delon, C., C. Galy-lacaux, D. Serça, B. Loubet, N. Camara, E. Gardrat, I. Saneh, R. Fensholt, T. Tagesson, V. Le Dantec, B. Sambou, C. Diop, and E. Mougin. 2017. Soil and vegetation-atmosphere exchange of NO, NH_3, and N_2O from field measurements in a semi arid grazed ecosystem in Senegal. Atmospheric Environment **156:**36–51.

Dentener, F. J. 2006. Global Maps of Atmospheric Nitrogen Deposition, 1860, 1993, and 2050. ORNL DAAC, Oak Ridge, Tennessee, USA. **DOI: 10.3334/ORNLDAAC/830**

Farmer, E., and J. Mbwika. 2012. End market analysis of Kenyan livestock and meat: a desk study. USAID microreport 184.

Fisher, J. B., K. P. Tu, and D. D. Baldocchi. 2008. Global estimates of the land–atmosphere water flux based on monthly AVHRR and ISLSCP-II data, validated at 16 FLUXNET sites. Remote Sensing of Environment **112:**901–919.

Galy-Lacaux, C., and C. Delon. 2014. Nitrogen emission and deposition budget in West and Central Africa. Environmental Research Letters **9:**125002.

Houlton, B. Z., Y.-P. Wang, P. M. Vitousek, and C. B. Field. 2008. A unifying framework for dinitrogen fixation in the terrestrial biosphere. Nature **454:**327–330.

IPCC 2019. 2019 Refinement to the 2006 IPCC Guidelines for National Greenhouse Gas Inventories. Calvo, E., K. Tanabe, A. Kranjc, J. Baasansuren, M. Fukuda, S. Ngarize, A. Osako, Y. Pyrozhenko, P. Shermanau, and S. Federici (eds.). IPCC, Switzerland.

Jarvis, S. C., and B. F. Pain. 1994. Greenhouse gas emissions from intensive livestock systems: their estimation and technologies for reduction. In White, D. H., S. M. Howden (eds.), Climate Change: Significance for Agriculture and Forestry 27-38. Springer.

Leenaars, J. G. B. 2013. Africa Soil Profiles Database, Version 1.1. A compilation of georeferenced and standardised legacy soil profile data for Sub-Saharan Africa (with dataset). Africa Soil Information Service (AfSIS) project. ISRIC-World Soil Information. DOI: 10.13140/2.1.2279.6489.

Levine, J. S., E. L. Winstead, D. A. B. Parsons, M. C. Scholes, R. J. Scholes, W. R. Cofer, D. R. Cahoon, and D. I. Sebacher. 1996. Biogenic soil emissions of nitric oxide (NO) and nitrous oxide (N_2O) from savannas in South Africa: the impact

of wetting and burning. Journal of Geophysical Research: Atmospheres **101**:23689–23697.

Meixner, F. X., Th. Fickinger, L. Marufu, D. Serça, F. J. Nathaus, E. Makina, L. Mukurumbira, and M. O. Andreae. 1997. Preliminary results on nitric oxide emission from a southern African savanna ecosystem. Nutrient Cycling in Agroecosystems **48:**123–138.

Otte, M. J., and P. Chilonda. 2002. Cattle and small ruminant production systems in sub-Saharan Africa. A systematic review. Livestock Information Sector Analysis and Policy Branch, FAO Agriculture Department. http://www.fao.org/3/a-y4176e.pdf

Otter, L. B., W. X. Yang, M. C. Scholes, and F. X. Meixner. 1999. Nitric oxide emissions from a southern African savanna. Journal of Geophysical Research: Atmospheres **104:**18471–18485.

Rees, R. M., M. Wuta, P. A. Furley, and C. Li. 2006. Nitrous oxide fluxes from savanna (miombo) woodlands in Zimbabwe. Journal of Biogeography **33:**424–437.

Robertson, G. P., and T. Rosswall. 1986. Nitrogen in West Africa: the regional cycle. Ecological Monographs **56:**43–72.

Rufino, M. C., E. C. Rowe, R. J. Delve, and K. E. Giller. 2006. Nitrogen cycling efficiencies through resource-poor African crop-livestock systems. Agriculture, Ecosystems and Environment **112**:261–282.

Satter, L. D., and R. E. Roffler. 1975. Nitrogen requirement and utilization in dairy cattle. Journal of Dairy Science **58:**1219–1237.

Schlesinger, W. H. 2009. On the fate of anthropogenic nitrogen. Proceedings of the National Academy of Sciences of the United States of America **106:**203-208.

Schlecht, E., F. Mahler, M. Sangaré, A. Susenbeth, and K. Becker. 1995. Quantitative and qualitative estimation of nutrient intake and faecal excretion of zebu cattle grazing natural pasture in semi-arid Mali. International Conference on Livestock and Sustainable Nutrient Cycling in Mixed Farming Systems of Sub-Saharan Africa, Addis Ababa, Ethiopia, 22-26 Nov 1993. International Livestock Centre for Africa.

Scholes, M. C., R. Martin, R. J. Scholes, D. Parsons, and E. Winstead. 1997. NO and N_2O emissions from savanna soils following the first simulated rains of the season. Nutrient Cycling in Agroecosystems **48:**115–122.

Seré, C., and H. Steinfeld. 1996. World livestock production systems: Current status, issues and trends. FAO Animal Production and Health Paper No. 127.

Siemens J. and M. Kaupenjohann. 2002. Contribution of dissolved organic nitrogen to N leaching from four German agricultural soils. Journal of Plant Nutrition and Soil Science **165:**675-681.

Syrstad, O. 1993. Evaluation of dual-purpose (milk and meat) animals. World Animal Review **77:**56–59.

Wachendorf C., F. Taube and M. Wachendorf. 2005. Nitrogen leaching from ^{15}N labelled cow urine and dung applied to grassland on a sandy soil. Nutrient cycling in Agroecosystems **73:**89-100.
